# A community-based cross-sectional study of dietary composition and associated factors among tuberculosis patients in China

**DOI:** 10.1038/s41598-024-53146-5

**Published:** 2024-02-01

**Authors:** Yan Zheng, Hui Chen, Canyou Zhang, Dongmei Hu, Fei Zhao, Wei Piao, Shujuan Li, Dabin Liang, Zongye Luo, Yueling Fan, Jianwei Gao, Jun Cheng, Dongmei Yu

**Affiliations:** 1 Center for Disease Control and Prevention of Jiulongpo District, Chongqing, China; 2 National Center for Tuberculosis Control and Prevention, Chinese Center for Disease Control and Prevention, Beijing, China; 3 Clinical Trial and Research Center, Beijing Hospital, Beijing, China; 4 National Institute for Nutrition and Health, Chinese Center for Disease Control and Prevention, Beijing, China; 5 Institute of Tuberculosis Control and Prevention, Guangxi Zhuang Autonomous Region Center for Disease Control and Prevention, Nanning, China; 6 Division of Tuberculosis Control and Prevention, Lingyun County Center for Disease Control and Prevention, Baise, China; 7 Shanxi Provincial Center for Disease Control and Prevention, Taiyuan, China

**Keywords:** Cancer, Immunology, Microbiology, Diseases, Health care, Health occupations, Medical research, Oncology

## Abstract

To determine the dietary structure and its associated factors of tuberculosis (TB) patients in the community. This cross-sectional study analysed the dietary intake of 300 TB patients in two impoverished counties in China. Food intake was collected by using food frequency and two consecutive 24-h dietary review (24hdr) methods. The dietary composition and dietary structure of TB patients were compared with China’s 2022 Dietary Reference Intake (DRIs) and the average reference value of dietary composition (ARC) in China in 2013. Binary logistic regression models were used to explore the factors associated with inadequate intake of animal food, insufficient protein and fat energy supply in patients with TB. The daily intake of various foods in TB patients was measured and the results were as follows: staple foods—median 372.12 g (interquartile range [IQR] 315.87 g); vegetables—median 200.00 g (IQR 205.55 g); fruits—median 20.22 g (IQR 36.82 g); animal foods—median 100.82 g (IQR 180.74 g); dairy products—median 0.00 g (IQR 0.00 g); nuts—median 17.10 g (IQR 29.75 g). The average daily intakes of vegetables, fruits, animal food, dairy products, soy and nuts were lower than those recommended by the DRIs (*P* < 0.01). Compared to women, men consumed more whole grains and mixed legumes, but less fruit. The dietary structures, including food and nutrient supply for energy, protein and fat, were significantly different in 300 patients compared with DRIs or ARC values. Inadequate rates of animal food intake were observed in 54.85% of men and 59.57% of women. Protein undersupply rates were 66.02% in men and 56.38% in women, while fat undersupply rates were 52.91% in men and 52.13% in women. The study revealed that being 18–49 years old, being the Han nationality, having less than 2 h of physical activity per day on average, and eating twice a day were risk factors for inadequate animal protein intake, protein energy deficiency and fat energy deficiency. TB patients from impoverished counties in China have inadequate intake of several food categories and insufficient protein and fat energy supply, correlating with multiple factors in socio-demographics, behavioral practices, and TB disease. To improve the nutritional status of TB patients, urgent public health actions, especially carrying out nutritional screening and evaluation once diagnosed, developing individualized nutritional support treatment plans, strengthening dietary nutritional health education and intervention, and advocating for enhanced nutritional support, should be taken.

## Introduction

In recent years, tuberculosis (TB) has been one of the leading causes of death from infectious diseases worldwide. China has been one of the 30 countries with the most TB burden^1^. Undernutrition increases the risk of TB, which can lead to malnutrition^2,3^. Malnutrition causes wasting, anemia, weight loss and decreased cellular immune function, and therefore plays a major role in activating latent TB infection^4^. Maintaining a reasonable dietary structure (one cup of milk a day and a regular diet of soy) and ensuring good nutrition are essential to promote disease recovery and sustain the life of TB patients^5,6^. However, nutritional status is often closely linked to the level of regional healthcare and socioeconomic status^7^. Currently, there are limited surveys and analyses on the dietary structure of TB patients in China. For instance, Chen Danping’s^8^ research on elderly TB patients and Sun Lin’s^9^ study on TB-combined diabetic patients relied heavily on dietary surveys conducted in a hospital setting. However, there is a lack of research on the dietary composition and structure of TB patients at community level. A cross-sectional study was conducted based on dietary surveys of pulmonary TB patients in two poverty-stricken counties in China to gain a better understanding of the nutritional status of pulmonary TB patients in poverty-stricken areas. The study results can be used to analyse the dietary composition and influencing factors of tuberculosis patients, providing a basis for formulating dietary guidance and care policies for such patients.

## Methods

### Ethical approval

This study was part of the project “Investigation of nutrition and diet of patients with pulmonary tuberculosis in poor areas in China^10^”. The study method was a cross-sectional survey, the survey protocol was approved by the Ethics Committee of the Chinese Center for Disease Control and Prevention (No: 201532), and informed consent was obtained from each participant for the on-site survey. In addition, the study was conducted in full accordance with the Helsinki Declaration.

### Sampling methods

Site selection criteria was developed based on three conditions considered. Firstly, the site must be poverty-stricken county. Secondly, it must be listed into the ‘Chinese Adult Chronic Disease and Nutrition Monitoring’ project in 2015. Thirdly, the performance of local routine TB control work was satisfied. Lin County in Shanxi Province and Lingyun County in Guangxi Zhuang were therefore chosen as the study sites according to this criteria.

Detailed sample size calculation methods have been described in other articles^10^. A prevalence of 6.7% was assumed for the general population and 25.0% for TB patients in poor areas. The sample size needed to detect differences in rates was then determined, with a probability of type I error set at 0.05, study efficacy estimated at 90%, and design effect set at 1. This resulted in a required sample size of 77 TB patients at each study site. Considering participants’ refusal, we expanded the sample size to 150 per study site, and the final sample size of TB patients was 300 totally.

### Participants

All adult patients (age ≥ 18 years) with active TB who were registered in Tuberculosis management information system from Nov 1st, 2015 through May 11th, 2017 were included into our survey according to the following inclusion criteria: (1) meeting the “Diagnosis Criteria of Tuberculosis” (WS 288-2008)^11^; (2) registered for the first time between November 1 of 2015 and April 30 of 2017 at the designated hospital; (3) classified as pulmonary according to “Classification of Tuberculosis” (WS 196-2001)^12^. And, those aged under 18 years old, having severe complications, being pregnant or lactating, and refusing to participate this survey were excluded from our survey.

### Investigation method

Patient face-to-face surveys and physical examinations were completed by county CDC staff with expertise in TB control and nutrition within 10 days of patient registration at designated hospitals and before anti-TB treatment. The surveys included four types of information: sociodemographic characteristics, behavioral habits, TB-related data and dietary status. The socio-demographic information included age, sex, ethnicity, education level, marital status, type of occupation, economic income, and family members. The behavioral and habitual information included alcohol consumption, smoking, physical activity duration, whether the food was all homemade, the frequency of meals per day, and vegetarian dietary habits. The TB-related information included height, weight, medical history, classification of treatments, results of chest imaging tests, sputum smears and culture results.

To assess dietary intake, we used two dietary habits questionnaires: the Food Frequency Questionnaire and the 24-h dietary recall method questionnaire. Trained investigators made a face to face investigation on the frequency and weight of each food item consumed on a daily, weekly, or monthly basis over the previous year. The survey’s food groups covered most dietary components, allowing for analysis of long-term intake of staples, vegetables, fruits, animal foods, dairy products, and legumes. The 24-h dietary recall method (24hdr) was used to evaluate patients’ food consumption over two consecutive days, one on a working day and the other on a weekend^13^. This included staple and non-staple foods, snacks, fruits, alcoholic beverages, and non-alcoholic beverages. Investigators utilised food models, containers and reference weights of various food sizes to assist patients in estimating food weight. As the food frequency method did not capture the intake of condiments, we employed the 24hdr to evaluate the intake of condiments and cooking oils over the preceding month to supplement the analysis of the patient’s dietary composition.

Specifically, the researchers performed a physical examination of the participants to collect data on the height and weight of the patients in the fasted state. Height was measured using a metal rod altimeter with an accuracy of 0.1 cm, while weight was measured using an electronic scale with an accuracy of 0.1 kg.

### Data processing

Based on the food frequency questionnaire, we calculated the average daily intake of six food groups in the past 12 months—staple foods, vegetables, fruits, animal foods, dairy products, legumes and nuts—and compared them with the recommended dietary intakes (DRIs) in the Chinese Dietary Guidelines for Chinese Residents (2022 edition)^14^ to assess the dietary composition of patients. Moreover, based on the 24hdr questionnaire, we calculated the patients’ energy and nutrient intake per food supply by reviewing the food composition^15^ and compared it with the average dietary composition ratio (ARC) in the Comprehensive Report on Nutrition and Health Monitoring of Chinese Residents 2010–2013^16^ to determine the patients’ dietary composition status.

According to the 2022 Chinese Dietary Guidelines, insufficient intake of animal-based foods is defined as insufficient intake of fish, poultry, eggs and meat. When the average daily intake of animal-based foods by individuals is less than 120 g, we believe that this is not enough. According to the results of nutritional status monitoring of Chinese residents in 2013, we believe that a protein energy supply component ratio less than 12.1% is not sufficient to meet the protein energy supply requirements of patients, and a fat energy supply component ratio less than 32.9% is not suitable for determining the fat energy supply of patients.

According to the national poverty standard (2011), individuals earning less than 2300 yuan annually were classified as low-income^17^, while those earning above this amount were divided into two groups with a cutoff point of 5000 yuan. Moreover, we classified smoking into three types. Smoking in the article specifically referred to smoking every day or occasionally during the week of the survey; former smoking referred to smoking in the past, but not currently; never smoking meant never smoking. For alcohol consumption, patients were considered to be alcohol drinkers if they had consumed at least one drink per week (25 g of spirits with an alcohol strength of 42° and above, 50 g of wine up to 42°, or one bottle of beer, 50 g of half a glass of yellow wine, or 150 g of wine) during the past month. We referred to the distribution of physical activity time for patients and divided them into three groups based on the cutoff at 2 and 5 h. Food sources were categorized into two types based on where the patient’s food was prepared in the past week: one all from homemade sources and the other not all from homemade sources. We classified BMI into three categories: underweight (BMI < 18.5 kg/m^2^), normal weight (BMI ≥ 18.5 kg/m^2^ and < 24.0 kg/m^2^), and overweight or obese (BMI ≥ 24.0 kg/m^2^)^18^.

### Statistical analysis

The dietary intake conditions are expressed as the mean (SD) and median (IQR). The Kolmogorov–Smirnov method was used to test the normality of the data. Continuous variables were compared using a one-sample or two-sample Student’s t-test (if the data were normally distributed); otherwise the Wilcoxon or Mann–Whitney rank sum test was used. A chi-square test was used to compare the differences in rates between the two gender groups. Binary logistic regression analysis was used to examine the association between inadequate animal food intake (low-protein dietary composition or low-fat dietary composition) and factors such as age, sex and income. A two-sided *P* value of less than 0.05 was considered to indicate statistical significance. The data were analysed by using SPSS software version 21.0.

## Results

### Characteristics of participants

Most of the patients were aged between 18 and 49 years, had a primary school education or less, were married, were employed and had an annual per capita income of at least 2300 yuan. Most did not consume alcohol or tobacco products and consumed mostly homemade meals three times a day. In addition, most of the patients had BMI values ranging from 18.5 to 23.9 and had no prior chronic conditions. Most patients had lesions in both lungs or more than two lung fields on chest X-ray imaging before treatment. We compared demographic, behavioral habit, and TB-related characteristics between male and female survey subjects (Table 1). The proportions of male patients who were employed, drank alcohol, smoked or had smoked, spent 5 h or more in outdoor activities, and ate twice a day were significantly greater than those of female patients (*p* < 0.05).

**Table 1:** Sociodemographic, behavioral and tuberculosis-related characteristics of 300 TB patients.

Characteristics	Total	Male	Female
	N (%)	n (%)	n (%)
Total	300	206	94
Sociodemographic characteristics			
Age group (years)			
18–49	161 (53.67)	106 (51.64)	55 (58.51)
50–64	86 (28.67)	66 (32.04)	20 (21.28)
65–85	53 (17.66)	34 (16.50)	19 (20.21)
Ethnic division			
The Han nationality	205 (68.33)	140 (67.96)	65 (69.15)
Minorities	95 (31.67)	66 (32.04)	29 (30.85)
Education			
Primary school and below	161 (53.67)	111 (53.88)	50 (53.19)
Junior middle school and above	139 (46.33)	95 (46.12)	44 (46.81)
Marriage			
Married	209 (69.67)	150 (72.82)	59 (62.77)
Unmarried	91 (30.33)	56 (27.18)	35 (37.23)
Ocupational status*			
Employed	178 (59.33)	137 (66.50)	41 (43.62)
Unemployed	122 (40.67)	69 (33.50)	53 (56.38)
Annual average income per person in the family (RMB)^a^			
< 2300	65 (21.67)	48 (23.30)	17 (18.09)
2300–4999	70 (23.33)	49 (23.79)	21 (22.34)
≥ 5000	85 (28.33)	59 (28.64)	26 (27.66)
Unknown	80 (26.67)	50 (24.27)	30 (31.91)
Behavioral and dietary habits characteristics			
Alcohol drinker*			
Nondrinker	181 (60.33)	116 (56.31)	91 (96.81)
Drinker	119 (39.67)	90 (43.69)	3 (3.19)
Cigarette smoker*			
Nonsmoker	160 (53.33)	69 (33.50)	91 (96.81)
Ex-smoker	87 (29.00)	85 (41.26)	2 (2.13)
Current smoker	53 (17.67)	52 (25.24)	1 (1.06)
Average daily outdoor activity time (hours)*			
< 2	79 (26.33)	44 (21.36)	35 (37.23)
2 ~ 4	106 (35.33)	73 (35.44)	33 (35.11)
≥ 5	115 (38.34)	89 (43.20)	26 (27.66)
Food source in the past week			
All homemade	252 (84.00)	175 (84.95)	77 (81.91)
Not all homemade	48 (16.00)	31 (15.05)	17 (18.09)
Daily meal frequency (times)*			
2	105 (35.00)	81 (39.32)	24 (25.53)
3	195 (65.00)	125 (60.68)	70 (74.47)
Vegetarian			
Yes	43 (14.33)	28 (13.59)	15 (15.96)
No	257 (85.67)	178 (86.41)	79 (84.04)
Received dietary guidelines and health education			
Yes	83 (27.67)	57 (27.67)	26 (27.66)
No	217 (72.33)	149 (72.33)	68 (72.34)
Tuberculosis related characteristics			
BMI (kg/m^2^)			
< 18.5	64 (21.33)	47 (22.82)	17 (18.09)
18.5-	201 (67.00)	132 (64.08)	69 (73.40)
≥ 24.0	35 (11.67)	27 (13.11)	8 (8.51)
Chronic medical history			
Yes	43 (14.33)	34 (16.50)	9 (9.57)
No	257 (85.67)	172 (83.50)	85 (90.43)
Therapeutic category			
Initial treatment	276 (92.00)	187 (90.78)	89 (94.68)
Retreatment	24 (8.00)	19 (9.22)	5 (5.32)
Sputum examination			
Pathogenic negative	253 (84.33)	174 (84.47)	79 (84.04)
Pathogenic positivity	23 (7.67)	15 (7.28)	8 (8.51)
Unknown	24 (8.00)	17 (8.25)	7 (7.45)
Lung lesion site			
Unilateral lung	137 (45.67)	88 (42.72)	49 (52.13)
Bipulmonary	163 (54.33)	118 (57.28)	45 (47.87)
Number of affected lung fields			
1	119 (39.67)	74 (35.92)	45 (47.87)
2	108 (36.00)	80 (38.83)	28 (29.79)
3–6	73 (24.33)	52 (25.24)	21 (22.34)
Cavity			
Yes	38 (12.67)	27 (13.11)	11 (11.70)
No	262 (87.33)	179 (86.89)	83 (88.30)

*The composition ratio of male and female was statistically different, *P* < 0.05.

^a^A total of 80 patients who refused or had missing data were excluded from this analysis.

### Various types of food intake

#### Intake of various types of food

Most of the patients had low food ranges in the previous 12 months and 48 h, and their main daily meals were whole grains, vegetables and animal meat. In addition, 19.00% of the patients did not eat fruit, 82.00% did not eat dairy products, 10.67% did not eat eggs and 14.00% did not eat meat, poultry, fish or other animal-derived foods during the previous 12 months. With respect to food intake, animal based food intake was similar to DRIs; and the intake of whole grains and mixed beans was greater than DRIs (*p* < 0.05); and the intake of vegetables, fruits, and dairy products was lower than DRIs (*p* < 0.05). In particular, both men and women had insufficient intake of dairy products, fruits, vegetables, potatoes, soybeans, and nuts (Table 2).

**Table 2:** Comparison of the intake of various foods/g (standard person. d)  − 1 among the surveyed population (food frequency method).

Food category	DRIs	Survey population median (IQR)	Males (n = 206)		Females (n = 94)		$\chi^{2}$	*P*值
			Median (IQR)	Below RNI (%)	Median (IQR)	Below RNI (%)		
Staple food	200–300	372.12 (315.87)^b^	438.96 (336.19)^a^	49 (23.79)	300.00 (227.58)^a^	37 (39.36)	7.657	0.006*
Whole grain and miscellaneous beans	50–150	300.00 (302.45)^b^	327.56 (310.22)^a^	3 (1.46)	190.87 (253.40)^a^	2 (2.13)	0.178	0.674
Tubers	50–100	15.23 (99.30)	15.23 (99.37)	119 (57.77)	16.33 (80.03)	57 (60.64)	0.219	0.639
Vegetables	300	200.00 (205.55)^b^	200.00 (202.36)	144 (69.90)	200.00 (228.77)	69 (73.40)	0.384	0.535
Fruits	200–350	20.22 (36.82)^b^	14.85 (37.73)^a^	202 (98.06)	21.37 (54.85)^a^	89 (94.68)	2.530	0.112
Animal food	120–200	100.82 (180.74)	101.92 (191.08)	113 (54.85)	96.29 (165.23)	56 (59.57)	− 0.785	0.433
Milk and milk products	300	0.00 (0.00)^b^	0.00 (0.00)	206 (100.00)	0.00 (0.00)	94 (100.00)	-	-
Legumes and nuts	25–35	17.10 (29.75)^b^	16.64 (30.07)	129 (62.62)	17.10 (26.75)	60 (63.83)	0.040	0.841

None of the data fit the normal distribution (*P* < 0.05).

*Statistical difference of chi-square test for comparison of inadequate food intake rates between the two gender groups.

^a^The Wilcoxon signed rank sum test was statistically different between the 2 groups.

^b^The results of the Wilcoxon signed rank sum test comparing sample intake with the upper or lower value of the RNI suggested a statistical difference.

#### Factors associated with insufficient intake of animal foods

The results of our binary logistic regression analysis suggested that multiple factors influence the animal food intake of TB patients. Factors such as: 18–49 years of age, from the Lincounty area, Han nationality, unmarried status, education level at middle school or above, unemployed status, family poverty status, alcohol consumption status, average physical activity duration less than 2 h, food intake not all from home preparation, two meals per day, self-reported vegetarian status, never heard of dietary guidelines, a negative sputum test, other chronic diseases, newly diagnosed tuberculosis, unilateral lung lesions, etc., were risk factors for inadequate food intake (*p* < 0.05) (Table 4).

### Dietary structure

#### Dietary composition

First, in terms of food energy sources, the main energy-supplying food for patients was whole grains (48.27% of total energy). The composition ratios of whole grains, tubers, edible oils, sugars, alcohol and other foods were lower than the ARC values (*p* < 0.05), while those of animal foods were higher than the ARC values (*p* < 0.05). In addition, compared with women, men had a greater proportion of whole grain energy supply, a lower proportion of edible oil, and a relatively lower rate of whole grain undersupply (*p* < 0.05) (Table 3).

**Table 3:** Comparison of dietary composition (%) of 300 patients by gender group (24-h dietary review survey method).

Food category	ARC	Survey population mean (SD) or median (IQR)	Males (n = 206)		Females (n = 94)		$\chi^{2}$	*P*值
			Mean (SD) or median (IQR)	Below ARC (%)	Mean (SD) or median (IQR)	Below ARC (%)		
Food sources of energy								
Whole grains^e^	53.1	48.27 (14.92)^d^	49.46 (16.03)^c^	124 (60.19)	45.67 (11.82)^c^	71 (75.53)	6.674	0.010*
Beans	1.8	1.40 (3.45)	0.98 (3.38)	120 (58.25)	2.08 (3.58)	44 (46.81)	3.411	0.065
Tubers	2.0	1.15 (5.89)^b^	1.45 (6.09)	109 (52.91)	0.90 (5.88)	52 (55.32)	0.150	0.698
Animal food	15.0	21.97 (35.20)^b^	21.78 (34.05)	91 (44.17)	22.75 (36.50)	42 (44.68)	0.007	0.935
Edible oil	17.3	7.91 (6.12)^b^	7.36 (5.77)^a^	193 (93.69)	9.89 (6.16)^a^	81 (86.17)	4.610	0.032*
Sugar	0.4	0.00 (0.00)^b^	0.00 (0.00)	204 (99.03)	0.00 (0.00)	93 (98.94)	0.006	0.940
Alcoholic drink	0.6	0.00 (0.00)^b^	0.00 (0.00)	204 (99.03)	0.00 (0.00)	94 (100.00)	-	1.000
Other foods	9.8	3.56 (4.36)^b^	3.57 (4.20)	188 (91.26)	3.25 (4.69)	81 (86.17)	1.806	0.179
Nutrient sources of energy								
Carbohydrate^e^	51.0	51.00 (13.71)	52.18 (14.55)	109 (52.91)	48.43 (11.32)	53 (56.38)	0.313	0.576
Protein^e^	12.1	11.51 (2.41)^d^	11.47 (2.12)	136 (66.02)	11.59 (2.95)	53 (56.38)	2.571	0.109
Fat^e^	32.9	30.82 (12.37)^d^	30.22 (12.98)	109 (52.91)	32.12 (10.84)	49 (52.13)	0.016	0.899
Food sources of protein								
Cereal crop	47.3	37.79 (43.52)	38.25 (44.75)	113 (54.85)	35.86 (38.78)	54 (57.45)	0.176	0.675
Beans	5.4	4.77 (11.89)	3.75 (11.75)	116 (56.31)	7.20 (11.94)	43 (45.74)	2.893	0.089
Animal food	30.7	35.24 (43.54)	33.93 (41.87)	95 (46.12)	42.30 (45.24)	38 (40.43)	0.847	0.357
Other foods^e^	16.6	14.97 (8.10)^d^	15.15 (8.06)	131 (63.59)	14.59 (8.23)	66 (70.32)	1.255	0.263
Food sources of fat								
Animal food	35.9	59.03 (71.24)^b^	61.53 (69.43)	82 (39.81)	54.59 (74.06)	37 (39.36)	0.005	0.942
Plant food	64.1	4.97 (71.24)^b^	38.47 (69.43)	124 (60.19)	45.41 (74.06)	57 (60.64)	0.005	0.942

None of the data fit the normal distribution (*P* < 0.05).

*There was a difference in below ARC-value rate between the 2 groups.

^a^The Wilcoxon signed rank sum test was statistically different between the 2 groups.

^b^The results of the Wilcoxon signed rank sum test comparing sample intake with the upper or lower value of the ARC suggested a statistical difference.

^c^The two gender group means were statistically different by t-test.

^d^The mean of the sample was statistically different from the ARC value by one-sample t-test.

^e^The data for this variable were normally distributed, while the data for the other variables were not.

Second, in terms of nutrient sources of energy, the energy composition ratio of carbohydrates was close to the ARC value, while the energy composition ratios of proteins and fats were lower than the ARC value (*p* < 0.05); and in terms of the proportion of energy supplied by the three main nutrients, there was no significant difference between male and female patients.

Third, regarding the food sources of protein, the protein composition ratios of cereals, legumes and animal foods were quite close to the ARC values, while the composition ratios of other food sources were higher than the ARC values (*p* < 0.05). In addition, there was no significant difference in the composition of protein sources between male and female patients.

Fourth, two counties had cases associated with regional dietary habits^5,19,20^. In Lin County of Shanxi Province, patients consumed pasta as their staple food, with 91.33%, 92.67%, 98.67%, and 100% having insufficient intake of animal food, vegetables, fruits, and milk, respectively. In Lingyun County, patients consumed rice as their staple food, with insufficient intake mainly of vegetables, fruits, and milk, at proportions of 49.3%, 95.3%, and 100%, respectively.

Finally, in terms of the food source of fat, there was a significant difference in the composition of fat in the patients compared to the ARC values. The percentage of animal foods was significantly greater than the ARC values (*p* < 0.05), and had no statistically significant differences between males and females overall.

#### Factors associated with deficiency of dietaryprotein supply

A significantly greater percentage of patients had protein energy deficiency (66.02% in men and 56.38% in women). Our binary logistic regression analysis of protein energy underprovision revealed that 6 factors, 18–49 years of age, from Lin County area, Han nationality, average daily physical activity time less than 2 h, 2 meals per day, and self-reported vegetarian status, were high risk factors for protein underprovision (*p* < 0.05) (Table 4).

**Table 4:** Factors associated with insufficient intake of animal derived foods、protein supply and fat supply using univariate regression analysis in 300 TB patients.

Associated factors	Insufficient intake of animal derived foods		Insufficient protein supply		Insufficient fat supply	
	OR (95%CI)	*P*	OR (95%CI)	*P*	OR (95%CI)	*P*
Socio-demographic factors						
Age						
18–49	1.00		1.00		1.00	
50–64	0.606 (0.356,1.030)	0.064	0.942 (0.543,1.635)	0.832	0.598 (0.352,1.014)	0.056
≥ 65	0.443 (0.236,0.833)	0.012	0.486 (0.259,0.913)	0.025	0.296 (0.153,0.571)	< 0.001
Area						
Lin county	1.00		1.00		1.00	
Linyun county	0.026 (0.013,0.051)	< 0.001	0.457 (0.283,0.738)	0.001	0.005 (0.002,0.014)	< 0.001
Gender						
Male	1.00		1.00		1.00	
Female	1.213 (0.739,1.990)	0.445	0.665 (0.404,1.096)	0.110	0.969 (0.595,1.579)	0.899
Ethnic division						
The Han nationality	1.00		1.00		1.00	
Minorities	0.076 (0.041,0.140)	< 0.001	0.528 (0.321,0.868)	0.012	0.039 (0.019,0.083)	< 0.001
Education						
Primary school and below	1.00		1.00		1.00	
Junior middle school and above	2.395 (1.495,3.837)	< 0.001	1.292 (0.805,2.071)	0.288	3.786 (2.338,6.132)	< 0.001
Marriage						
Married	1.00		1.00		1.00	
Unmarried	3.704 (2.120,6.472)	< 0.001	1.593 (0.939,2.701)	0.084	3.357 (1.966,5.734)	< 0.001
Occupation						
Employed	1.00		1.00		1.00	
Unemployed	1.273 (0.798,2.029)	0.312	0.796 (0.495,1.281)	0.348	1.102 (0.694,1.748)	0.681
Annual average income per person in the family (RMB)^a^						
< 2300	1.00		1.00		1.00	
2300–4999	0.217 (0.086,0.547)	0.001	1.037 (0.498,2.161)	0.923	0.308 (0.134,0.706)	0.005
≥ 5000	0.073 (0.030,0.179)	< 0.001	0.550 (0.279,1.083)	0.084	0.127 (0.057,0.283)	< 0.001
Behavioral factors						
Alcohol drinker						
Non-drinker	1.00		1.00		1.00	
Drinker	2.649 (1.645,4.264)	< 0.001	0.889 (0.551,1.434)	0.630	0.462 (0.289,0.741)	0.001
Smoking status						
Non-smoker	1.00		1.00		1.00	
Ex-smoker	0.560 (0.330,0.950)	0.032	0.833 (0.489,1.417)	0.499	0.437 (0.257,0.745)	0.002
Current smoker	0.623 (0.333,1.166)	0.139	1.896 (0.939,3.827)	0.074	0.625 (0.335,1.168)	0.141
Average daily physical activity time						
< 2 h	1.00		1.00		1.00	
2 ~ 4 h	0.092 (0.037,0.230)	< 0.001	0.539 (0.293,0.990)	0.046	0.234 (0.113,0.482)	< 0.001
≥ 5 h	0.044 (0.018,0.110)	< 0.001	0.976 (0.528,1.805)	0.938	0.066 (0.032,0.138)	< 0.001
Food preparation						
All home production	1.00		1.00		1.00	
Not all home production	2.371 (1.198,4.694)	0.013	1.210 (0.631,2.323)	0.566	2.000 (1.045,3.827)	0.036
Daily habitual dining frequency						
2 times	1.00		1.00		1.00	
3 times	0.282 (0.167,0.477)	< 0.001	0.426 (0.252,0.721)	0.001	0.438 (0.268,0.716)	0.001
Vegetarian						
Yes	1.00		1.00		-	
No	42.992 (5.829,317.093)	< 0.001	0.235 (0.096,0.576)	0.002	-	-
Heard of the dietary guidelines for Chinese resident						
Yes	1.00		1.00		1.00	
No	5.207 (2.802,9.674)	< 0.001	0.708 (0.414,1.213)	0.209	0.037 (0.015,0.096)	< 0.001
Tuberculosis related information						
BMI						
< 18.5	1.00		1.00		1.00	
18.5–23.9	1.156 (0.657,2.034)	0.614	1.226 (0.689,2.181)	0.488	1.209 (0.688,2.123)	0.509
≥ 24.0	1.324 (0.574,3.053)	0.511	1.158 (0.496,2.703)	0.735	0.842 (0.369,1.924)	0.683
Chronic medical history						
Yes	1.00		1.00		1.00	
No	0.114 (0.049,0.266)	< 0.001	0.882 (0.455,1.710)	0.71	0.223 (0.105,0.472)	< 0.001
Therapeutic category						
Initial treatment	1.00		1.00		1.00	
Retreatment	0.095 (0.028,0.325)	< 0.001	0.466 (0.201,1.079)	0.075	0.341 (0.137,0.848)	0.021
Sputum examination						
Pathogenic negative	1.00		1.00		1.00	
Pathogenic positivity	0.486 (0.205,1.152)	0.101	0.904 (0.377,2.170)	0.822	0.397 (0.163,0.971)	0.043
Not checked	0.126 (0.042,0.381)	< 0.001	0.969 (0.408,2.301)	0.943	0.196 (0.071,0.541)	0.002
Lung lesion site						
Unilateral lung	1.00		1.00		1.00	
Bipulmonary	0.491 (0.308,0.784)	0.003	0.856 (0.534,1.373)	0.519	0.374 (0.234,0.598)	< 0.001
Number of affected lung fields						
1	1.00		1.00		1.00	
2	0.609 (0.354,1.046)	0.073	0.930 (0.539,1.604)	0.794	0.565 (0.329,0.970)	0.038
3–6	0.275 (0.149,0.508)	< 0.001	0.712 (0.391,1.296)	0.266	0.154 (0.080,0.296)	< 0.001
Cavity						
Yes	1.00		1.00		1.00	
No	1.930 (0.969,3.845)	0.062	1.128 (0.562,2.265)	0.735	2.383 (1.168,4.863)	0.017

Intake above RNI/ARC was assigned as 0, intake below RNI/ARC was assigned as 1.

^a^The sample size of analysis of annual household income was 235.

#### Factors associated with a deficient dietary fat supply

Similarly, 52.91% of men and 52.13% of women had a high percentage of insufficient fat energy. According to the binary logistic regression analysis of inadequate fat energy supply, 18–49 years of age, Lin County area, Han nationality, junior high school education, unmarried status, poor family, no alcohol consumption, no smoking, less than 2 h of physical activity per day on average, 2 meals per day, food not all made at home, heard of dietary guidelines, history of chronic disease, a negative sputum test, invasion of only 1 lung field, and absence of cavitation were elevated risk factors (*p* < 0.05) for developing inadequate fat supply (Table 4).

## Discussion

### Malnutrition and dietary intake in patients with tuberculosis

We found that the prevalence of low body weight malnutrition in patients with tuberculosis was 21.33%, which is higher than that of the general adult population in China and significantly lower than the prevalence of low body weight malnutrition in patients with tuberculosis found in studies in Ghana, Ethiopia and Bangladesh^21–25^. Most of the patients’ diets were based on 3 categories—whole grain staples, vegetables and meat—suggesting a monotonous diet for TB patients, which could easily lead to nutrient imbalance and malnutrition. In addition, patients were generally deficient in dairy and animal foods, suggesting that inadequate protein intake and frequency of consumption may be the main causes of protein malnutrition in patients.

We found that the problem of insufficient animal protein intake among patients was particularly pronounced in Lin County, suggesting that there may be some regional differences, and these findings are consistent with those of other studies in China^26^. Patients in Lin County mainly consumed potatoes, flour products and meat, just like the locals. A total of 39.33% were from poor families, 28.67% self-reported to be vegetarians, 28.00% had a habit of drinking alcohol, and 46.67% ate only two meals per day. These social, behavioral, and dietary habits may be the main reasons for the insufficient protein intake of patients in Lin County. We also found that the risk of inadequate protein intake was greater in patients who drank alcohol and ate out, which was similar to the findings of Zhou et al.^27^. However, we did not find any difference in the rate of insufficient protein intake between pathology-positive and pathology-negative patients, which may be related to too few pathology-positive sputum test results.

Moreover, most patients had inadequate intake of vegetables and fruits, which may explain the inadequate intake of vitamin C in 63.1% of patients in previous studies^10^. Vitamin C, an essential nutrient for cell-mediated immunity, not only has some bacteriocidal activity, but it also promotes the supply of immune defenses to the various cells of the immune system and protects the host from the damage caused by reactive oxygen species and reactive nitrogen radicals produced by TB infection^4,29,30^. Li et al.^24^ used high-dose vitamin C (1 g/d) injection or oral adjuvant treatment for tuberculosis, and the percentage of sputum-negative individuals increased significantly after 3 months in the vitamin C supplementation treatment group, which also confirmed that vitamin C plays an essential role in anti-tuberculosis autoimmunity. Therefore, more nutritional interventions should be recommended for patients with insufficient vitamin C intake^4,29,30^.

We also found a lower risk of protein or fat malnutrition in patients with more severe tuberculosis lung infections, with relapse. This may be because consumptive diseases such as tuberculosis prompt patients to ensure their nutritional intake, or it may reflect the effectiveness of previous nutritional health guidance from physicians. Medical staff can intervene to actively modify the diet of patients with inadequate protein supplies and abnormal amino acid levels, prompting them to supplement them with exogenous protein. Between 2004 and 2011, four randomized controlled clinical trials examined the effects of protein-energy supplementation on tuberculosis treatment, and the results were relatively consistent: patients who received nutritional interventions gained between 2 and 6 kg^31–34^. To avoid severe illness and death from TB, to shorten the course of treatment and to improve the quality of patients’ health, we also recommend strengthening the protein and energy supply of patients, to ensure the protein supply of patients with malnutrition, history of previous chronic disease, re-treatment, and severe lung disease and to minimize the harm of TB.

As a result, the risk of animal food intake deficiency varies among patients with various characteristics such as region, age, ethnicity, education level, marital status and economic status. To ensure that TB patients have an adequate supply of food and energy, according to the World Health Organization Dietary Guidelines for TB Patients and the Chinese Dietary Pagoda (2022) and taking the differences in food availability and dietary habits in different regions and seasons into account, we suggest that a list of recommended diets be developed for TB patients in different regions and seasons and with different social characteristics. For example, in Lin County, Shanxi Province, patients were advised to increase their daily intake of pork, eggs, dairy products, vegetables and fruits; and in Lingyun County, Guangxi Zhuang Autonomous Region, patients were advised to increase their daily intake of soybeans, dairy products and fruits. Moreover, nutritional subsidies or free nutritional breakfasts are recommended for patients from poor families, and nutritional risk screening and nutritional counseling should be carried out in a timely manner to improve the relevance and effectiveness of dietary interventions for TB patients.

### The dietary structure of TB patients differs from that of the general population

There are fewer domestic studies on the dietary structure of TB patients. In 2018, Chen et al.^8^ reported that the dietary structure of elderly TB patients in Shanghai was unreasonable. Similarly, Zhao et al.^26^ reported insufficient intake of dairy products, vegetables, fruits, and eggs among TB patients in Lingyun and Hepu counties in Guangxi. Our study yielded similar results. Our previous study revealed that 87.38% of male patients and 82.98% of female patients had inadequate energy intake^10^. In our study, we also found that the structure of the patient’s dietary energy supply differed from that of the general population. Patients consumed a lower percentage of energy from cooking oil and soy than did the general population, and a greater percentage of energy was from animal foods.

The nutrient composition of energy among TB patients also differed from that in the general population, with a lower proportion of protein and fat in their energy supply. The patients’ protein intake mainly came from whole grains and animal foods, accounting for 45.51% and 32.16%, respectively. This proportion of animal protein intake was similar to that in the general population but did not reach the recommended value of 50% high-quality protein intake for TB patients as recommended by nutrition experts^3,35–37^. The dietary composition of fat in patients was also different from that in the general population, and the proportion of animal fat was greater in these patients than in the general population.

The type and adequate amounts of high-quality lean proteinare very important. We need to focus on dietary patterns where patients have an inadequate intake of animal foods or protein. According to the “Expert Consensus on Nutritional Assessment and Nutritional Support Treatment for Critically Ill Patients with Tuberculosis”, we recommend that physicians in tuberculosis clinics carry out nutritional screening and nutritional assessment before and during treatment and formulate nutritional support programs for patients with newly diagnosed or newly managed active tuberculosis. Adequate intake of high-quality protein should be ensured^35,38,39^.

The study also revealed differences in protein energy supply among patients from different regions, ages, ethnic groups, hours of physical activity and dietary habits. In particular, a vegetarian diet and fewer meals significantly increase the risk of inadequate protein and fat energy supplies. For example, patients in Lin County had a greater risk of protein and fat energy deficiency than did those in Lingyun County, and ethnic minorities had a lower risk of protein and fat energy deficiency than did Han Chinese individuals. These results suggest that there are clear regional and ethnic differences in the dietary composition of TB patients, which may be related to regional dietary habits. To improve the dietary structure of patients, we suggest that during the diagnosis, treatment and follow-up of TB patients, medical staff should provide reasonable dietary advice according to the characteristics of region, ethnicity and disease development, especially to advise patients to increase their intake of high-quality protein.

### TB patients have insufficient awareness of nutritional knowledge

We found that patients who were familiar with the Chinese dietary guidelines had a lower risk of inadequate animal food intake. However, they did have a greater risk of insufficient protein and fat intake. This suggests that we need to pay more attention to educating people about nutritional knowledge and its effects on health. The World Health Organization recommends providing dietary counseling for patients receiving antituberculosis treatment and those suffering from malnutrition^40^. Considering that individuals with higher education levels are at greater risk for inadequate animal food intake, we recommend the development of tailored nutrition plans and targeted nutrition education campaigns based on varying levels of education, lifestyle habits, physical activity levels, and other individual characteristics. Additionally, as vegetarianism, smoking, drinking alcohol and other behaviors have been linked to insufficient protein and fat energy supplies from animal sources, it is important to address these factors in any comprehensive nutritional intervention. We suggest that patients actively promote smoking cessation, alcohol cessation, a balanced diet and other healthy lifestyles during health education.

### Advantages and limitations

Our study is of great practical value for guiding the future community management of TB patients. First, the study is community-based rather than healthcare facility-based, which has strong reference value for community-based patient management. Second, we used a comprehensive and systematic nutritional survey to analyze the dietary structure of TB patients and compared it with the 2013 Chinese Dietary Nutrient Reference Intake and the 2022 Chinese Dietary Guidelines. We explored the factors related to dietary structure, such as sociodemographic, behavioral, and disease-related factors, which laid a foundation for the development of policies to improve patient nutrition and health.

This study has several limitations. First, the food frequency method has its own shortcomings, which may include recall bias leading to the omission of part of the food information^32^. The 24hdr method does not involve weighing ingested food, and there may be bias in food weight; moreover, the study analyzed only the recent dietary structure of patients and did not assess the long-term dietary structure status of patients. Second, the sample size of 300 people in this study was slightly insufficient, multifactorial analysis of factors related to dietary structure could not be performed due to insufficient sample size, single-factor correlations may have confounding effects, and the sample size of the nutritional survey of community tuberculosis patients needs to be increased in the future to obtain additional research data.

## Conclusion

In this study, we found that in poor areas of China, TB patients had insufficient intake of dairy products, vegetables, and fruits. Generally, protein and energy intake is insufficient. The intake of animal source food and the dietary structure of the energy supply of patients are affected by many factors, such as region, education level, age, frequency of meals, and food source. To carry out effective treatment for tuberculosis patients, it is necessary to take public health action, carry out nutritional screening and nutritional evaluation in a timely manner, formulate individualized nutritional support treatment plans for patients, strengthen dietary nutrition health education and intervention, and advocate government nutritional support for poor patients to ensure the nutritional supply of tuberculosis patients.

### Supplementary Information

Supplementary Information.

## Data Availability

The datasets generated and/or analysed during the current study are available in the [supplementary file–zydata].
